# Factors Contributing to Satisfaction with Changes in Physical Function after Orthopedic Surgery for Musculoskeletal Dysfunction in Patients with Cerebral Palsy

**DOI:** 10.1371/journal.pone.0154749

**Published:** 2016-05-02

**Authors:** Yasuaki Kusumoto, Osamu Nitta, Atsushi Matsuo, Kenji Takaki, Tadamitsu Matsuda

**Affiliations:** 1 Department of Physical Therapy, Faculty of Health Sciences, Tokyo University of Technology, Ota-ku, Tokyo, Japan; 2 Department of Physical Therapy, Faculty of Health Sciences, Tokyo Metropolitan University, Arakawa-ku, Tokyo, Japan; 3 Department of Orthopedic, Minamitama Orthopedic Hospital, Machida, Tokyo, Japan; 4 Department of Rehabilitation, Minamitama Orthopedic Hospital, Machida, Tokyo, Japan; 5 Department of Physical Therapy, Faculty of Health Sciences, Uekusa-Gakuen University, Chiba, Chiba, Japan; Iranian Institute for Health Sciences Research, ACECR, ISLAMIC REPUBLIC OF IRAN

## Abstract

**Background:**

The recognition of required treatments for cerebral palsy (CP) patients, including orthopedic surgery, differs according to region. This study was performed to identify factors associated with satisfactory changes in physical function after orthopedic surgery.

**Methods:**

358 patients were selected for the questionnaire survey. The following information was collected: gender, primary disease, age of initial surgery, total procedural count, operated sites, satisfaction of postoperative rehabilitation frequency, ideal amount of postoperative rehabilitation sessions per week, frequency of voluntary home training per week, satisfaction of the timing of surgery and the current satisfaction with the changes in physical function after the orthopedic surgery. We classified the patients into the satisfied and dissatisfied group according to satisfactory changes in physical function after the surgery. We performed unpaired t-tests and chi-square tests to determine the variables that differed significantly between the groups. Variables with a p value of <0.2 were included in the multivariate logistic regression analysis.

**Results:**

The logistic model was revised and summed up to two potential predictors of postsurgical satisfaction with physical function: satisfaction with the frequency of postoperative rehabilitation sessions and the orthopedic surgery of the hip (distinction hit ratio, 75.4%).

**Conclusions:**

This study demonstrated that the frequency of postoperative rehabilitation and history of hip surgery seemed to be related to the satisfaction with the changes in physical function after orthopedic surgery.

## Introduction

The orthopedic surgery for cerebral palsy (CP) has progressed over the years, conducted with various techniques to lower the rate of patient morbidity [1–4]. Families of CP patients whom underwent orthopedic surgery experienced lack of given information and effective interconnection between related facilities. Therefore, there is an urgent need for systematically securing the smooth adaptation of interdisciplinary consensus, globally as well as locally, by the related facilities, with aims to further empower families and healthcare professionals who are involved in this complex transferring process [5]. The recognition of required medical treatment for CP patients, including orthopedic surgery, differs according to region in Japan. These multiple recognition differences can influence orthopedic surgeries, which sometimes lead to unfavorable surgery outcomes and differences in regional protocols. Children and families require continuous interventions and services which are to be offered by multiple facilities [6]. There is a severe lack of interconnection between the hospitals and facilities regarding postoperative rehabilitation in Japan. This comes as a huge disadvantage for patients receiving treatments.

Orthopedic surgeries have several purposes such as pain relief, improvement of motor function and deformity. A study evaluating the satisfaction of surgical treatment performed on CP children with musculoskeletal dysfunction, reported that improvement of motor function led to improvement of quality of life and increased the opportunity for participating in outdoor sports activities [7,8]. Another study evaluating satisfaction of changes in physical function after surgical treatment, reported that the reasons involved improvement of gait, pain relief, and the improvement of range of motion, among others. However, whether the location of the surgical site influenced the current satisfaction of changes in physical function remains unclear. Previous studies have not identified factors which showed high correlation with the current satisfaction of changes in physical function. The information of medical treatment related to the post operation intervention concerning multi-institutional services that the patient and family need is plainly insufficient.

The purpose of this study was to identify the factors associated with satisfaction with the changes in physical function after orthopedic surgery in CP patients.

## Methods

### Participants

A total of 745 patients who underwent surgery at an Orthopedic Hospital in Tokyo from April 2005 to December 2009 were included in this study. 440 questionnaires were collected from the participants, and patients with CP indicated as the primary disease were selected. 38 patients with apoplexy, 17 with congenital diseases besides CP, and 27 participants with uncompleted questionnaires were excluded from this study. Based on the responses of the questionnaire, a total of 357 patients with CP as the primary disease (response rate: 48.1%) were included in study.

### Procedures

We developed the original questionnaire based on previous studies to investigate postoperative rehabilitation and satisfaction concerning changes in physical function [6–8]. The preliminary version of the original questionnaire included 13 question items. Content validity of the questionnaire was established using written feedback from 10 clinicians. Clinicians were defined as those having over five years of evaluating experience with CP patients (experience range 5–47y, mean: 19y). The clinicians consisted of three physicians and seven physical therapists. Participants were asked to check either ‘agree’, ‘disagree’, or ‘undecided’ for each question item. If they chose ‘disagree’ or were unable to decide, they were asked to provide an explanation and suggest changes. Frequencies of each response were recorded for all question items. When more than 80% of agreement was observed, each question item was accepted to the questionnaire without amendments. As a result, two items (satisfaction with changes in physical function after surgery, and the frequency of preoperative rehabilitation) were excluded and 11 contents were revised and accepted. Explanations and suggestions associated with these contents were critically examined. Amendments to the preliminary version of questionnaire were made, conducting a pretest with 20 subjects. The randomly recruited subjects were CP patients whom underwent orthopedic surgery (age of initial surgery; 7–48 year). After two months, we retested with the same subjects and the consistency of results were confirmed by paired t-tests and chi-square tests. No significant differences were found. So we used the preliminary version of questionnaire for the final version.

The questionnaire (please refer to S1 Questionnaire) was conducted from 1 to 22 February in 2010. Briefing papers were mailed to the participants. We had requested the questionnaires to be sent back upon agreement to participate in this study. When patients were either too young or presented hardship to communicate, the next of kin or legally authorized representative consented on behalf of the participants. This research received approval from the ethical review board of Minamitama Orthopedic Hospital (approval number. 001).

The survey using the questionnaire collected the following information from the patients: name of the person entering the data, the patient’s gender, primary disease, age of initial surgery, number of procedures, surgery sites, satisfaction with the frequency of postoperative rehabilitation sessions, ideal frequency of postoperative rehabilitation sessions per week, frequency of voluntary training at home per week, satisfaction with the timing of the surgery and the current satisfaction with changes in physical function after the surgery. These satisfactions were graded using a 5-point scale (very satisfied, satisfied, neither, dissatisfied, or very dissatisfied). Each patient was classified into the satisfied group (very satisfied and satisfied) or dissatisfied group (neither, dissatisfied, very dissatisfied).

### Statistical analysis

In order to investigate the factors contributing to the satisfaction with changes in physical function with as few variables as possible, we initially performed the unpaired t-tests and chi-square tests to determine which variables differed significantly between the satisfied and the dissatisfied group. The number of procedures, age at the time of the initial surgery, ideal number of postoperative rehabilitation sessions and frequency of voluntary training at home per week were used as independent variables and satisfaction with the changes in physical function was used as the dependent variable. The presence of association with the variables were assessed by the unpaired *t*-tests. On the other hand, gender, surgery sites, satisfaction with the frequency of postoperative rehabilitation sessions, and timing of the surgery were used as independent variables, while satisfaction with the changes of physical function was used as a dependent variable. The associations were assessed by chi-square tests. Secondly, those variables with a p value of <0.2 were used as independent variables and the current satisfaction with changes in physical function were used as a dependent variable. These associations were assessed by multivariate logistic regression analyses with forward stepwise selection (likelihood ratio). Statistical analyses were performed using SPSS (IBM, for Windows version 19.0). A *P value of* ≦0.05 was considered to imply statistical significance.

## Results

Table 1 shows the results from unpaired *t*-tests and chi-square tests. According to the satisfaction of changes in physical function, there were 253 participants in the satisfied group and 105 participants in the dissatisfied group. The results of unpaired *t*-tests presented age to be a significant factor at the time of the first surgery. The patients in the satisfied group had a lower average age than the dissatisfied group (*P* = 0.005). The results of chi-square tests suggested that, satisfaction with the frequency of postoperative rehabilitation sessions and the surgery site at the hip joint were also significant factors. The frequency of surgery at the hip joint (*P* = 0.012) and satisfaction with the frequency of postoperative rehabilitation sessions (*P* = 0.000) were significantly higher in the satisfied group than the dissatisfied group. And p values of surgery sites at the neck, knee joint and ankle were lower than 0.2.

**Table 1:** Characteristics of satisfied and dissatisfied groups according to the current satisfaction with changes in physical function after orthopedic surgery.

	All(n = 358)	Satisfied group(n = 253)	Dissatisfied group(n = 105)	p value
**Gender(male / female:n)**	204 / 154	148 / 105	56 / 49	0.369
**Age of initial surgery (year)**	19.3(2~72)	17.5±15.5	23.5±19.1	0.005*
**Number of procedures (times)**	2.2±1.2	2.2±1.2	2.2±1.3	0.974
**Surgery site (multiple answers allowed)**				
**Neck (n)**	62	38	24	0.125
**Abdominal (n)**	10	8	2	0.511
**Back (n)**	16	11	5	0.863
**Shoulder (n)**	46	31	15	0.601
**Elbow (n)**	53	38	15	0.859
**Rist (n)**	29	21	8	0.830
**Hand (n)**	16	10	6	0.463
**Hip (n)**	236	177	59	0.012*
**Knee (n)**	106	81	25	0.116
**Ankle (n)**	137	104	33	0.086
**Foot (n)**	12	10	2	0.327
**Satisfaction with the frequency of postoperative rehabilitation sessions (satisfied group / dissatisfied group:n)**		163 / 90	31 / 74	0.000*
**Ideal frequency of postoperative rehabilitation session in week (times)**	3.9±1.7	3.9±1.7	4.0±1.8	0.577
**Frequency of voluntary training at home per week (times)**	2.6±1.5	2.6±1.5	2.6±1.5	0.937
**Satisfaction with the timing of the surgery (satisfied group / dissatisfied group:n)**		153 / 100	58 / 47	0.359

Age of initial surgery and number of procedures, ideal frequency of postoperative rehabilitation session per week, and frequency of voluntary training at home per week were assessed by unpaired *t*-tests. Gender of patients, surgery site, satisfaction with the frequency of postoperative rehabilitation sessions, and satisfaction with the timing of the surgery were assessed by chi-square tests.

*:p < .05

Table 2 shows the results of multivariate logistic regression analysis. There were six independent variables: age at the time of the initial surgery, satisfaction with the frequency of postoperative rehabilitation sessions, the surgical sites of neck, hip, knee joint and ankle. Consequently, the logistic regression analysis had selected satisfaction with the frequency of postoperative rehabilitation sessions (odds ratio, 5.138; 95% confidence interval, 3.052–8.652) and the surgery site at the hip joint (odds ratio, 0.462; 95% confidence interval, 0.273–0.780) as contributing factors. Moreover, the distinction hit ratio was 75.4%. Therefore, these results indicate that the patients with a higher frequency of postoperative rehabilitation sessions or those who underwent surgery at the hip joint experienced a higher degree of satisfaction.

**Table 2:** Results of multivariate logistic regression analysis of factors contributing to the satisfaction of changes in physical function.

	regression coefficient	standard error	wald	degree of freedom	significance probability	odds ratio	95% confidence interval of odds ratio	
**Degree of satisfaction with the frequency of postoperative**	1.637	0.266	37.911	1	0.000	5.138	3.052	8.652
**hip joint of surgery site**	-0.773	0.267	8.364	1	0.004	0.462	0.273	0.780
**Constant**	-2.836	0.441	41.325	1	0.000	0.059		

Filter: Satisfaction group = 0, Dissatisfaction group = 1, distinction hitting ratio:75.4%.

## Discussion

The purpose of this study was to clarify contributing factors to the satisfaction with changes in physical function after orthopedic surgery in CP patients. We first performed bivariate analysis to determine which variables differed significantly between the satisfied and dissatisfied groups. The following variables with p values set at <0.2 were included in the multivariate logistic regression analysis: age of initial surgery, satisfaction with the frequency of postoperative rehabilitation sessions and surgery sites, which were the neck, hip joint, knee joint and ankle. Consequently, the frequency of postoperative rehabilitation sessions and surgery site at the hip joint were found to be predictive factors of positive outcomes in patients’ satisfaction.

One study examining the outcome of a single-event multilevel surgery in ambulant CP children reported that parental concerns over insufficient rehabilitation might undermine parental satisfaction [9]. Because our study also presented similar results, where satisfaction with the frequency of postoperative rehabilitation sessions was related to satisfaction with changes in physical function, we summarized that patients and guardians required adequate postoperative rehabilitation. The differences in ideal frequency of postoperative rehabilitation sessions per week between the satisfied and the dissatisfied groups were not significant. Both groups suggested that postoperative rehabilitation should ideally be performed four times a week. Furthermore, recrudescence of deformity and symptoms, limitation of playing activity, slow-healing of surgical wounds from incision were found to be unfavorable postsurgical outcomes [9,10]. The social environment after discharge proved to affect the satisfaction of the orthopedic surgery [9]. Therefore, maintaining a good environment for prolonged periods of post-surgery rehabilitation and a favorable environment for both work and play activity was important.

In general, the purposes of conducting orthopedic surgeries on the lower extremity are to improve standing and gait function [1,2]. Upper extremity surgeries are targeted in improving upper limb mobility, to bear rolling, crawling operability and skilled motor maneuvers [3,4]. The purposes of trunk and neck surgeries are to improve as well as prevent cervical spondylotic myelopathy and relieve excess retroflexion [1]. Hip orthopedic surgeries are typically performed to relieve pain, improve hip dislocation, mat activity, standing and gait function. It was reported that orthopedic hip surgeries led to an improvement of torque in knee extension after four weeks, allowing selective muscle release interventions [11], and lower extremity surgeries led to improvement in gross motor function [12]. In previous studies, improvements in walking ability showed the most significant correlation with parental satisfaction [9]. Thus, it can be considered to be as a contributing factor to satisfaction with one of the extracted factors. Moreover, hip orthopedic surgeries were necessary to achieve pain relief, improvement of hip dislocation, standing and gait function.

The mean age of the participants in the satisfied group at the time of the first surgery was significantly lower than in the dissatisfied group. The purposes of orthopedic surgery has a tendency of improving or achieving crawling, standing and being able to walk because infants and children grow and develop. For adults, on the other hand, orthopedic surgery is aimed at relieving pain for secondary disabilities such as cervical spondylotic myelopathy, low back pain and improving gait functions. The satisfied group included many infants and children who experienced improvements of physical ability after surgeries. However, the dissatisfied group included many adults with whom the surgeries did not yield desired outcomes. The age of initial surgery was a significant factor according to the unpaired *t*-test, but was not significant after the multivariate logistic regression analysis. When we performed the multivariate logistic regression analysis to analyze the satisfaction of the post operation intervening frequency and the factor of conducted surgical area of the hip, by observing the difficultly for some of the patients in maintaining a relatively functional physical condition (maximum of 5 years post-operation) until the time of the conducted questionnaire, we comprehended that it might have been a contributing factor to influence the satisfactory results of the surgery. For the Japanese interdisciplinary knowledge to lead to satisfying outcomes, it was important to acknowledge that it might be difficult for patients to maintain a relatively functional physical condition on a long-term basis.

## Supporting Information

S1 Questionnaire Questionnaire about orthopedic surgery. (PDF)
