# Supplementary material for: Factors Contributing to Satisfaction with Changes in Physical Function after Orthopedic Surgery for Musculoskeletal Dysfunction in Patients with Cerebral Palsy
Source: PLoS One. 2016 May 2;11(5):e0154749. doi: 10.1371/journal.pone.0154749 (PMC4852902; doi:10.1371/journal.pone.0154749)
Supplement: S1 Questionnaire — (PDF) [file pone.0154749.s001.pdf]

# Questionnaire about orthopedic surgery

Q1. Who are you entering this questionnaire?

・Person　　　　　　・Gardians

Q2. Please select the gender of the patient.

・Male　　　・Female

Q3. What is his/her primary disease?

・Cerebral palsy　　・Stroke　　　・Else（　　　　　　　　　　　　　）

Q4. How old was he/she at the time of the first surgery?

______________________year__

Q5. How many times has he/she had ever surgeries?

______________________times__

Q6. Circle the surgery sites that he/she had ever undergone.

Neck・Abdominal・Back・Shoulder・Elbow・Rist・Hand・Hip・Knee・Ankle・Foot

Q7. Please circle those that apply.

The satisfaction with the frequency of postoperative rehabilitation sessions is [ very satisfied ・ satisfied ・ neither ・ dissatisfied ・ very dissatisfied ].

Q8. How many times were ideal frequency of postoperative rehabilitation sessions in a week?

____________________ times

Q9. How many times were frequency of voluntary training at home per week?

____________________ times

Q10. Please circle those that apply.

The satisfaction with the timing of the surgery is [ very satisfied ・ satisfied ・ neither ・ dissatisfied ・ very dissatisfied ].

Q11. Please circle those that apply.

The current satisfaction with physical function change after orthopedic surgery is [ very satisfied ・ satisfied ・ neither ・ dissatisfied ・ very dissatisfied ].
